# Metallosis after Exchange of the Femoral Head and Liner following Ceramic Acetabular Liner Dissociation in Total Hip Arthroplasty with a Modular Layered Acetabular Component

**DOI:** 10.1155/2016/5301451

**Published:** 2016-08-25

**Authors:** Tomoya Takasago, Tomohiro Goto, Keizo Wada, Daisuke Hamada, Toshiyuki Iwame, Tetsuya Matsuura, Akihiro Nagamachi, Koichi Sairyo

**Affiliations:** Department of Orthopedics, Institute of Biomedical Sciences, Tokushima University Graduate School, Tokushima, Japan

## Abstract

The type of bearing material that should be used in revision surgery after the failure of ceramic-on-ceramic total hip arthroplasty (THA) remains controversial. In the case of ceramic fracture, the residual ceramic particles can cause consequent metallosis when metal implants are used for revision THA. On the other hand, in the case of THA failure without ceramic fracture, revision THA with a metal femoral head provides satisfactory results. We report an unusual case of progressive osteolysis due to metallosis that developed after revision THA for ceramic liner dissociation without a liner fracture performed using a metal femoral head and polyethylene liner. The residual metal debris and abnormal pumping motion of the polyethylene liner due to the breakage of the locking system or the aspherical metal shell being abraded by the ceramic head seemed to be the cause of the progressive osteolysis.

## 1. Introduction

Alumina ceramic components have been used in total hip arthroplasty (THA) for over 30 years. These implants were introduced to reduce wear and to increase long-term survivorship. A modular layered acetabular component, consisting of an alumina ceramic liner housed in an ultra-high-molecular-weight polyethylene shell (ABS liner) that is held in a titanium alloy metal shell (AMS HA shell), was developed in Japan (Kyocera, Kyoto, Japan) for use in alumina ceramic-on-ceramic THA. The main limitations of this component are the risk of ceramic liner fracture and failure of the ceramic liner fixation system [1–3]. Moreover, revision THA after ceramic liner fracture can be problematic. For example, in revision THA using a metal femoral head, metallosis may occur as a consequence of abrasive wear caused by ceramic particles deposited in periarticular tissues [4–6]. On the other hand, in the case of ceramic liner dissociation due to the breakage of the locking system between the socket and liner without ceramic fracture, revision THA with a metal femoral head may provide satisfactory results [7]. We report here an unusual case of progressive osteolysis due to metallosis that developed after revision THA for ceramic liner dissociation without liner fracture in THA with a modular layered acetabular component.

## 2. Case Presentation

**Figure 1:**
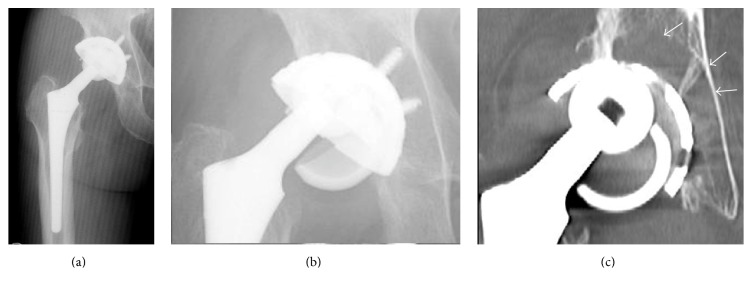
A postoperative anteroposterior radiographic image showing primary THA with alumina-on-alumina bearings on the right side for the treatment of secondary osteoarthritis due to developmental dysplasia of the hip (a). A plain radiograph and a computed tomography (CT) image, obtained 10 years after primary THA, showing the liner's dissociation from the metal shell along with retroacetabular osteolysis (white arrows) (b and c).

**Figure 2:**
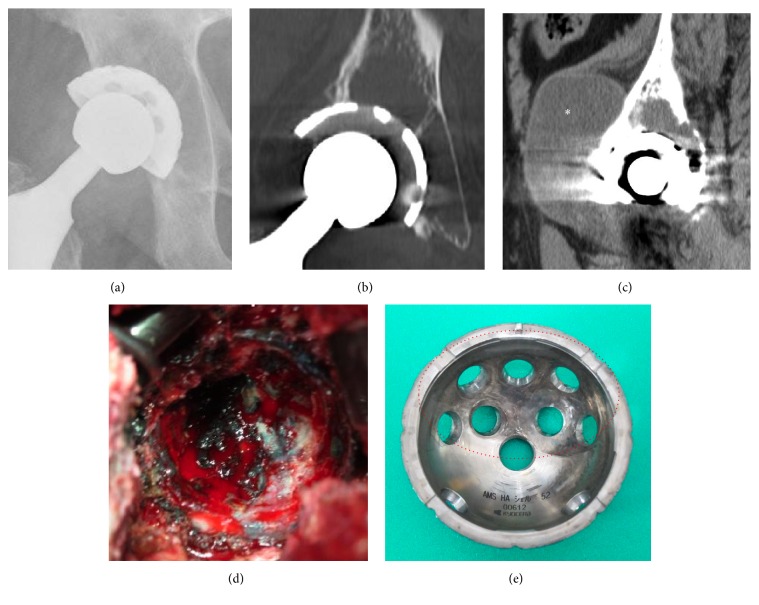
An anteroposterior view of plain radiograph (a) and a CT coronal image (b) showing the expanding retroacetabular osteolysis. A pseudotumor at the anterior aspect of the right hip (asterisk) connected to the hip joint was observed in a CT sagittal image (c). Pigmentation of metallic debris inside the osteolytic lesion and around the joint capsule and acetabulum was seen intraoperatively (d). A photograph of the retrieved cup (e) showing abrasion inside the metallic shell connecting to polyethylene liner (dotted circle).

**Figure 3:**
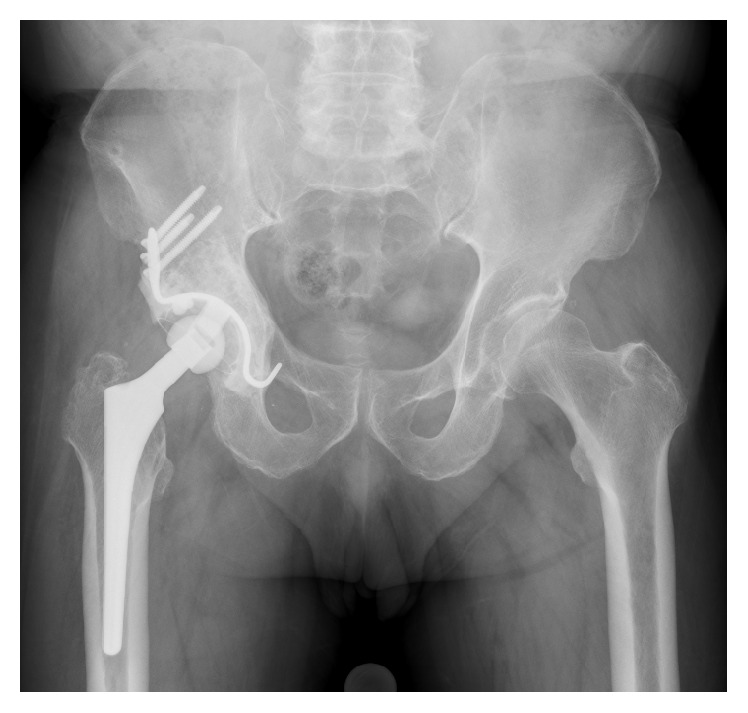
An anteroposterior plain radiograph, obtained 4 years after the rerevision surgery, showing acetabular reconstruction performed with a Kerboull-type acetabular reinforcement device and bulk structural allograft.

A 64-year-old man underwent primary THA with alumina-on-alumina bearings on the right side for the treatment of secondary osteoarthritis due to developmental dysplasia of the hip by posterolateral approach (Figure 1(a)). The implants used were a modular layered acetabular component, a titanium alloy stem, and a ceramic head (AMS HA shell with ABS liner 52 mm, PerFix HA stem size 11, and alumina ceramic head 28 mm with +4 mm offset; Kyocera, Kyoto, Japan). The postoperative course was uneventful. At the age of 74, 8 years after the last follow-up, he presented with right hip pain and crepitus during walking. Plain radiographs and computed tomography (CT) images of the hip revealed failure of the acetabular component, including liner dissociation from the metal shell, and osteolysis behind the cup was observed in DeLee and Charnley zones I and II (Figures 1(b) and 1(c)). At the time of the first revision surgery, no fracture or damage was observed at the alumina ceramic liner, ceramic head, or neck of the stem, but the polyethylene rotation prevention mechanism was observed to have failed. Pelvic osteolysis and black metallic debris behind the metal socket were observed through the screw holes. The cup and stem were well fixed and hence left in situ. We removed the granulomatous tissue and metallic debris around the cup as much as possible, and the backside debris was also debrided using screw holes. The liner and head were replaced with a highly cross-linked polyethylene and cobalt-chrome alloy head (910 MX liner CP, PHS metal ball 28 mm with +4 mm offset; Kyocera, Kyoto, Japan). At two years and five months following the first revision surgery, pain and swelling were noted over the right groin. Plain radiographs and CT images confirmed expanding retroacetabular osteolysis in all 3 zones and an intrapelvic pseudotumor, which is classified as fluid filled type, connecting to the right hip joint (Figures 2(a), 2(b), and 2(c)). Black synovial fluid was observed by puncture of the mass. A diagnosis of pseudotumor due to metallosis was made and the patient was referred to our hospital. Subsequently, rerevision THA was performed for managing the retroacetabular osteolysis and metallosis with direct lateral approach. Intraoperative findings included black pigmentation of metallic debris within the osteolytic lesions and around the joint capsule and acetabulum (Figure 2(d)). Bone ingrowth was noted only at the periphery of the cup, and it was easily removed by utilizing an explant device. The osteolytic lesions, synovial tissue, and joint capsule were debrided to remove the metal debris to the maximum possible extent. Acetabular reconstruction was performed with a Kerboull-type acetabular reinforcement device and bulk structural allograft (Figure 3). The well-fixed femoral stem was left in situ using a titanium sleeve on the trunnion with a new ceramic head (KT plate 480010, standard socket liner 44 mm, and alumina ball 28 mm with standard offset; Kyocera, Kyoto, Japan). There were no metal or ceramic particles on the polyethylene liner articulation, and little damage on the cobalt-chrome alloy head was observed by scanning electron microscopy of the retrieved cup, polyethylene liner, and head. The inside of the metallic shell connecting to polyethylene liner had abraded, possibly resulting in direct contact with the alumina ceramic head before the first revision surgery performed for dissociation of the ceramic liner (Figure 2(e)). Furthermore, abnormal pumping movement between the polyethylene liner and the metallic shell and slight deformity and abrasion in the liner locking system were observed on inspection of the retrieved cup. A loose locking mechanism and the liner malseating to the metallic shell caused this abnormal pumping movement. After rerevision surgery, the postoperative course was uneventful. At the latest follow-up, that is, 4 years after the surgery, the patient was able to walk without an aid and was independent in all activities of daily living, with no evidence of osteolysis or loosening of the implant.

## 3. Discussion

The type of bearing that should be used after a fracture in ceramic-on-ceramic THA remains controversial, and there are no prospective studies since few patients suffer this complication. Revision THA with metal-on-polyethylene pairing is not feasible. Despite performing radical synovectomy and thorough washout of the hip joint, it may not be possible to remove all of the minute ceramic fragments following such ceramic fracture. While ceramic is a material with higher rigidity than metal, severe damage to the metal head and the polyethylene liner may occur due to wear from residual ceramic particles, resulting in early failure as metallosis [8, 9]. On the other hand, placing a new ceramic head on an undamaged trunnion has been shown to be effective. Hannouche et al. reported implantation of standard ceramic heads onto well-fixed stems in 61 cases of revision surgery with no ceramic head fractures after 7 years of follow-up [10]. However, it is difficult to exclude microscopic trunnion damage during such revision surgery, and implanting a ceramic head on a damaged trunnion carries a certain risk of ceramic head fracture or earlier failure. In recent years, the use of a trunnion adaptor or sleeve, which ensures a pristine interface between ceramic and metal, has become common as a new option for minor trunnion damage. Jack et al. reported excellent survival rate and function after utilizing a sleeve on a used/damaged trunnion together with a ceramic head [11].

In the case of acetabular component failure without ceramic fracture, revision THA with a metal femoral head can be a reasonable treatment option because of the absence of residual ceramic particles in the joint [7]. In the present case, metallosis occurred unexpectedly after the first revision THA using a metal femoral head and a polyethylene liner although there was no ceramic fracture. At first, we speculated that ceramic particles existed within the articular surface leading to metallosis. However, the retrieved cup and head showed no metal or ceramic particles on the polyethylene liner articulation, and little damage was noted on the cobalt-chrome alloy head by scanning electron microscopy. In addition, no obvious damage at the trunnion of the well-fixed stem was observed intraoperatively. Maloney et al. reviewed 35 revision THA cases for pelvic osteolysis after the primary cementless THA with a porous-coated acetabular component and reported that none of the osteolytic lesions had progressed after revision surgery involving the exchange of the liner [12]. In contrast, in the present case, retroacetabular osteolysis continued to expand after replacement of the liner and head. In the first revision THA, where the well-fixed shell was left in place, metal debris generated by direct impaction between the metallic shell and alumina ceramic head after liner dissociation might have remained. Moreover, an abnormal pumping movement between the polyethylene liner and the metallic shell was observed on inspection of the retrieved cup, which may have contributed to the abnormal expansion of the osteolysis. Several authors have suggested that the pumping movement between the polyethylene liner and the metallic shell may pump fluid and particles from the space between the liner and the shell through the screw holes to the retroacetabular bone [13–15]. Walter et al. documented two versions of the polyethylene pumping mechanism, namely, diaphragm pumping characterized by deformation of a noncongruent liner suspended at the rim of the shell and piston pumping characterized by pistoning of the liner in and out of the shell, both of which probably coexist in vivo [16]. In the present case, these two pumping mechanisms might have occurred, leading to the flow of fluid in and out of the joint through the screw hole. As a result, some metal debris remained inside the retroacetabular osteolytic lesion, leading to expansion of the osteolysis along with diffusion around the periacetabular soft tissue, for example, into the intrapelvic space.

At the rerevision surgery, we considered that removal of the cup and radical debridement of the osteolytic lesion and pseudotumor were necessary. We performed rerevision THA using a reinforcement plate with bulk structural allograft. This technique is also useful for recovering bone stock and restoring the leg length and an adequate hip center [17].

We have presented a unique case of progressive osteolysis after revision surgery comprising exchange of the femoral head and liner due to ceramic acetabular liner dissociation in THA with a modular layered acetabular component. It is important to consider the possibilities of damage to the metal shell as well as the polyethylene liner locking mechanism in the case of ceramic articulation failure treated by such exchange of the liner and femoral head. In such cases, the removal of the entire implant and complete debridement of the metal debris may be required for reconstruction surgery.
